# Examining obesity kuznets curve in the United States from the perspective of globalization and biocapacity

**DOI:** 10.1016/j.heliyon.2023.e19569

**Published:** 2023-09-01

**Authors:** Gloria Nnadwa Alhassan, Andrew Adewale Alola, Festus Victor Bekun

**Affiliations:** a Department of Clinical Pharmacy, Faculty of Pharmacy, Cyprus International University, North Cyprus, Turkey; b CREDS-Centre for Research on Digitalization and Sustainability, Inland Norway University of Applied Sciences, Norway; c Faculty of Economics, Administrative and Social Sciences, Nisantasi University, Istanbul, Turkey; d Faculty of Economics Administrative and Social sciences, Istanbul Gelisim University, Istanbul, Turkey; e Adnan Kassar School of Business, Lebanese American University, Beirut, Lebanon

**Keywords:** Obesity, Income, Globalisation, Natural capital, United States

## Abstract

Given the recent rise in the adult obesity prevalence in the United States, the central and state-level governments and health agencies in the country are considering appropriate measures. Further motivation for this investigation stems from the United Nations Sustainable Development Goals (UNSDG-3, 8, and 13), that highlights the need for sustainable health for all, sustainable decent economic growth amidst environmental sustainability. Driven by this motivation, this study investigates the validity of Obesity Kuznets curve (OKC) in the United States over the experimental period of 1975–2016. In addition, this study illustrates the (mediating) role of globalisation and biocapacity in controlling the prevalence of obesity in the United States. While the study established the validity of obesity Kuznets curve, especially in the short run, it found an inverted U-shaped relationship between globalisation and obesity for the United States. This interprets that a significant reduction in wealth-related health issues is achievable with increased (socioeconomic and political) globalisation policy amidst improved (socioeconomic) welfare of the Americans. Moreover, biocapacity showed a desirable impact on obesity since the short- and long-run relationship with a respective elasticity of 0.02 and 1.86 is negative and statistically significant. In general, this study puts forward policy from the perspective of socioeconomic and political globalisation and domestic welfare measures across the country.

## 1 Introduction

Healthy living is a sensitive and important subject across the globe. Given the importance of health to human, health delivery targets for both developed and developing countries have increasingly been linked to the socioeconomic aspects. For instance, an individual's income distribution capacity makes an impact on the health status, thus making health-related issues increasingly associated with the upsurge in income inequalities, especially in the low-income countries. Importantly, several health-related challenges such as the prevalence of obesity are both peculiar to the advanced developed and developing countries as reported in Refs. [[1], [2], [3], [4]]. Obesity is considered an epidemic that needs to be controlled and prevented, especially in younger children, considering its major effect on the economy, mortality rate, and life expectancy. Specifically, the World Health Organisation (WHO) classifies overweight and obesity as an unusual accumulation of excessive fat tissues resulting in unhealthy quality of life. Out of all the several methods used in measuring the fat tissue in the body, body mass index (BMI) is the simplest and most popular method used medically and by populations in measuring the rate of adipose tissue.

The obesogenic environment is enthused by inexpensive calorie-dense food, disrupting technologies and lack of replacement of physical activity. This epidemic issue globally reached more than four-fold rise from 4% to 18% during the period 1975–2016. Similarly, in 2017, a mortality rate of over 4 million was recorded each year due to overweight or obesity as documented by the global burden of disease, in both developed and developing countries. In 2016, 39% of adults were overweight while the obese rate for children under five was 13% (38.3 million children) by 2019 [5,6]. Being a risk factor to advance health issues such as stroke, cardiovascular diseases, liver cirrhosis, high blood pressure, cancer, diabetes, and osteoarthritis, obesity is categorised as a non-communicable disease (NCD) Risk Factor Collaboration (NCD-RisC). Specifically, many studies attribute the menace of obesity and diabetes globally as twin epidemic [7,8]. The NCD-RisC recorded a fast increase of unnecessary accumulation of excessive fat among males and females in the period 1975–2014 from 3.2% to 10.8% and 6.4%–14.9%, respectively [9].

As reported by the Centres for Disease Control and Prevention (CDC), there is at least 20% prevalence of obesity in each state and territory of the United States [10]. Additionally, the CDC report further implies that one out of five children and more than one out of three adults suffers from obesity in the United States, thus making it a common, serious and costly ailment in the country. Moreover, in 2017, according to the updated report by the Organisation for Economic Co-operation and Development (OECD), the United States recorded the highest adult obesity rate among the OECD countries with 40% and Japan is lowest with 4.2% [11]. The OECD further opined that obesity rate is highest in the order of the United States, Mexico, and New Zealand among the OECD regions. Worse yet, it is projected that obesity rates will show a higher increase by 2030. Meanwhile, part of the Sustainable Development Goals (SDGs) 2030 agenda as laid out by all United Nations Member States in 2015 promotes “Good Health and Well-being, No Poverty, Responsible Consumption and Production and Decent Work and Economic Growth” [12,13].

Sequel to the theory of Simon Kuznets’ curve hypothesis [14], studies have shown that health inequality and income progression also have a non-direct link known as the health Kuznets curve encompassing the obesity Kuznets curve [[15], [16], [17]]. With the extension of health Kuznets curve, extant studies have further illustrated the income-weight-related health issues, thus establishing the obesity Kuznets curve [18,19]. Given that health is a normal good, people can shift over time, thus leading to a decline in the rate of obesity and its secondary complications. Following this line of thought, studies have presented evidence of the role of income, education, and socioeconomic aspects in reducing the prevalence of obesity for different cases [16,[20], [21], [22]]. Moreover, in the 21st century, it is irresistible to examine the nexus of globalisation and obesity given the increasing integration of economies across the globe which now account for high penetration of multinational companies (such as the food and beverages, and fast-food restaurants).

Following the aforementioned motivation, the current study seeks to close existing gap in the literature by employing the integration of social, economic and political aspects of the globe (globalisation) alongside the earth's biological capacity in examining the existence of obesity Kuznets curve in the United States. The study offers a novel and significant importance from different perspectives. Foremost, the role of (socioeconomic and political) globalisation and the square of globalisation in explaining the prevalence of obesity are controlled, thus establishing whether the existence of an inverted U-shaped relationship between globalisation and obesity hold. This study is aimed at revealing whether the increasing pace of globalisation pose any further danger or benefit to obesity prevalence rate in the United States. It is well noted in the extant literature on the theme with root stemming from the Simon Kuznets curve hypothesis. However, there is a paucity study that focuses on OKC in the context of United State. Empirical study abound that lend support for positive impact of increase income level on health while contrasting evidence also exist as outlined in the literature section of the present study in section 2. Furthermore, empirical evidence corroborates that economic growth gradients in body mass index when there is excessive fat tissue threshold while development is tied to heathier body mass index and healthy choices for the United States. Additionally, the study further contributes to the existing literature by explaining the role of the earth's biological capacity or natural capital in mitigating the menace of obesity prevalence in the United States. Insights into this sort of relationship is timely importance to the United State given the prevalence of obesity cases recorded in the study period. Additionally, policy blueprint can be glean from this study for the rest of the countries on the world. Studies of this sort are arguably of scientific importance and contribution to the health energy economics literature for the related stakeholders.

Notably, other sections of the study are orderly presented as follows. The existing and related studies are explained in section two while the data and methodological approaches are detailed in section three. The finding is discussed in section four and detail the summary of the investigation with policy insight in section five.

## 2 Literature review

A limited body of similar studies has been carried out using the Simon Kuznets curve and other methods in analysing the impact of income on health and a link between good health, lifestyle, health expenditure, and growth in developed and developing countries. Several studies have proven that age and gender have a significant and positive relationship with income and health for a single and cross-countries cases [[23], [24], [25]]. In the studies, findings support the positive impact of an increase in income on health while contrasting evidence has also been illustrated. For instance Ref. [26], provides evidence of a negative growth gradient in body mass index when there is an excessive fat tissue threshold and that developments are correlated with healthier BMI values and healthy choices for the United States. The study used unconditional quantile regression (UQR) estimator for the analysis with the national health and nutrition examination survey (NHANES) which revealed that, in the last 35 years, poor income countries have never had a significantly high rate of obesity status.

[27] also deduced a non-linear link between economic growth levels and obesity rates among women from about 244 Demographic and Health Surveys (DHS) for 1991–2009 for more than 56 countries. Similarly [16], supported this by conducting an Obesity Kuznets curve analysis of white females. The link between country per capital income and obesity rate is positive, as demonstrated by Refs. [28,29]. Meanwhile, in time of economic crises, low-income countries potentially experience a decline in obesity rate, thus are less likely to have weight-related health problems. However [30], used pooled cross-sectional data to analyse the effect of economic on health during the 1990s in Cameroon. The result revealed that child malnutrition increases during the country economic crises, meaning that underweight is the reverse side of overweight; this implies that obesity will decline in developing countries undergoing recession crises. Similarly [31], investigated the link between economic crises and health and concluded that in time of rising unemployment rate in the US, the obesity rate declined perceptibly.

In addition [32], conducted a study using a spline regression analysis to investigate more than 175 countries. The result revealed that Gross Domestic Product (GDP) is positively linked to BMI, with about US$ 3000 per capita but with little or no relationship beyond these levels. Meanwhile [33], carried out a study to examine the hypothesis that posits that obesity is triggered by economic growth for the examined case in the period 1930–1983 by using gender and age as major criteria. In contrast to their study [34], examined 20 countries with top high obesity rates for the period 1991–2016, By using the autoregressive distributed lag (ARDL) method, the result revealed a visibly negative impact of economic growth on obesity rate and good health for some of the examined countries. A similar result was seen from study carried out by Ref. [35] for 38 countries from 1991 to 2010. The study used multiple models for the analysis and incorporated national economic measures and standard such as foreign stock index, GDP and means tariffs.

Moreover [36], used a time pilot method to examine the relationship between the increase in obesity rate and income growth in South Africa for the period between 1994 and 2014. The result reveals that inequality is statistically associated with gender obesity. A higher increase in the obesity rate among women than men is observed while per capita GDP is directly linked to obesity in both genders. In addition [37], conducted a study using the obesity Kuznets curve model to analyse the link between country income and an increase in obesity rate. The study indicates a positive relationship between income and obesity in developing countries facing recession and negative for rich developed countries. Meanwhile, low-calorie food consumption and investing in a good healthy lifestyle are positively linked to a country's economic sustainability and life expectancy [38].

While there is a well-documented literature on the EKC such as [[39], [40], [41]]. However, there is paucity of study on obesity Kuznets curve that highlights the nexus between obesity and economic growth especially through the channel of globalization. From the study of [34], there is statistically significsnt evidence that globalization plays a pertinent role of impacting demand for energy and much more environmental sustainability. Furthermore, while considering health effect from an environmental lens, an increase in emission level is critical in contributing to global temperature that brings about several sedentary lifestyles in contributing to obesity issues. Although the connection between public health and environmental degradation grounds, through the occurrence of obesity is explained by much emission which translate into reduced physical activities with little to less energy requirement with low energy demand persons stand a high risk of obesity. Furthermore, human-anthropogenic activities increase GHGs emission which increases insomnia that results from the decrease in blood PH and in turn contributes to increase body weight gain [42].

Although, the role played by the wave of globalization on the increase obesity i.e., global demand for more intake of calorie and biocapacity is apparently limited in the United State in the extant literature. Following the literature reviewed, the contributions of this study to are in several ways:

First, the present study incorporates all facet of globalization i.e., social, economic, and political aspect of globalization to the obesity Kuznets curve (OKC) argument for the case of USA a nation highly plagued with obesity. The variable for the present study are apparently carefully chosen as it align with the United Nations Sustainable Development Goals (SDGs) agenda to be achieved by 2030 which is lacking in most studies.

Second, the present study also advances the OKC literature by incorporating the wave of globalization and income to the mix with the dynamic of the effect of fluctuation health-environment. This dimension is mainly neglected on the OKC literature especially for USA.

Thirdly, our study uses autoregressive distributed lag (ARDL) method that simultaneous renders the short and long run analysis over the examined variables.

## 3 Data and empirical approach

### 3.1 Data

In this study, the dataset (which was collected from free online sources) employed for the case of the United States covers an annual experimental period of 1975–2016. The choice to the period is due to limited data availability. However, the description, unit of measurement and sources of the data are presented in Table 1. Additionally, the graphical illustration of the associated trend of the dataset is provided in Fig. 1 (a - d) in the appendix.

**Table 1:** Variable description, statistics, correlation, and stationarity estimation.

Panel A							
Variable	Unit of measurement	Source	Mean	Maximum	Minimum	SD	J-Bera
OBSEISTY	Body Mass Index (BMI)	WHO	13.776	21.400	5.500	5.324	3.706
INCOME	Gross Domestic Product Per capita (Constant 2010 USD)	WDI	31143.48	57927.52	7801.457	15220.42	2.815
GLOBAL	Index that measures economic, social and political dimensions	KOF GI	72.769	82.225	61.067	7.009	3.682
BCP	Biologically productive area measured in global hectares	GFN	4.002	4.605	3.513	0.347	3.285

Correlation Matrix (**Panel B**)	OBSEISTY	INCOME	GLOBAL	BCP
OBSEISTY	1.000			
INCOME	0.991^a^	1.000		
GLOBAL	0.992^a^	0.977^a^	1.000	
BCP	−0.945^a^	−0.941^a^	−0.941^a^	1.000

Unit Root (**Panel C**) Level First difference								
Variables	ADF(c)	ADF (c + t)	KPSS(c)	KPSS (c + t)	ADF(c)	ADF (c + t)	KPSS(c)	KPSS (c + t)
OBSEISTY	−3.855^a^	−3.647^b^	0.801^a^	0.129^c^	−2.922^c^		0.238	0.200^b^
INCOME	1.153207	−2.664827	0.811^a^	0.198^b^	−4.341^a^	−4.436^a^	0.249	0.082
GLOBAL	−2.200	−1.136	0.793^a^	0.183^b^	−6.280^a^	−6.410^a^	0.201	0.087
BCP	−1.200	−5.818^a^	0.780^a^	0.103^c^	−10.314^a^	−5.979^a^	0.333	0.298^a^

Note: KOF GI, WHO, WDI and GFN are respectively KOF Globalisation Index, World Health Organisation, World Development Indicator, and Global Footprint Network databases.

To provide guidance for the direction of the investigation, Fig. 1 offers graphical information about the procedure of the empirical analysis. Specifically, this procedure begins with the estimation of the descriptive statistics (in procedure A) to the main estimation technique (in procedure B), and end with series of diagnostics tests (in procedure C). Importantly, the descriptive statistics (Panel A of Table 1) illustrate that the employed variables are normally distributed (given the insignificant values of the Jarque-Bera statistics). Importantly, the income per capita (GDP per capita) deviates maximally while the deviation experienced by biological capacity is the minimum among the examined variables. Concerning the correlation, statistical evidence (from Panel B in Table 1) suggests that obesity is positively triggered by income and globalisation, while biological capacity negatively correlates with obesity.

**Fig. 1:**
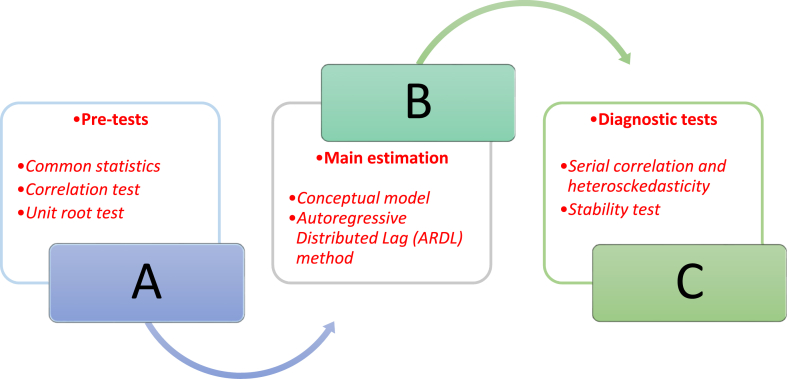
Procedure of the empirical analysis.

In proceeding towards examining the periodic impact of income and globalisation on the trend of obesity in the United States, the stationarity of the dataset is investigated. The Kwiatkowski, Phillips, Schmidt and Shin (KPSS) [43] stationarity test and the Augmented Dickey-Fuller (ADF) unit root approach described by Refs. [44,45] were employed. Accordingly, the result (Panel C of Table 1) revealed that all variables are stationary at most after first difference, thus paving way for the estimation of the evidence of cointegration.

### 3.2 Conceptual model

In line with [16,21] concerning the relationship between income and obesity, the current study explores this dimension for the case of the United States. Moreover, the current approach incorporates globalisation in the obesity-income model such that the impact of the square of income and the square of globalisation on obesity are both examined concurrently (equation (1)). Then, the logarithmic values are employed so as to reduce the concern of heteroscedasticity and report the result in elasticity form as shown in equation (2).

(1) $$OBESITY=f\left(INCOME,{INCOME}^{2},GLOBAL,{GLOBAL}^{2}\right)$$

(2) $${LnOBESITY}_{t}=\alpha +\beta_{1}\;\mathrm{Ln}\;{INCOME}_{t}+\beta_{2}{LnINCOME}_{t}^{2}+\beta_{3}{LnGLOBAL}_{t}+\beta_{4}\;\mathrm{Ln}\;{GLOBAL}_{t}^{2}+\varepsilon_{t}$$

The intercept and slope parameters are respectively α and $\beta_{i}$, where *i* = 1, 2, 3, and 4.

Furtherance to the concept of Kuznets curve and based on economic intuition, it is expected that $\beta_{1}>0$ while $\beta_{2}<0$. Similarly, the signs of $\beta_{3}$ and $\beta_{4}$ could be positive or negative such that the U-shaped or Inverted U-Shaped nexus between obesity and globalization is validated. Subsequently, the empirical estimation is conducted in order to establish the aforementioned relationships. To this end the following hypotheses will be investigated

(i) Does higher income level induce obesity in the study area?
(ii) To what extent is globalisation impact on United State obesity level?
(iii) Is there a statistical difference between obesity and biocapacity?

However, a robustness estimate that incorporates biological capacity (BCP) in the obesity Kuznets curve model is employed, such that

(3) $$OBESITY=f\left(INCOME,{INCOME}^{2},GLOBAL,BCP\right)$$

#### 3.2.1 Empirical method

Given that the maximum order of integration of the variables is I (1) i.e., stationary after first difference, the appropriateness of the ARDL by Ref. [46] is brought to bear. The ARDL is also considered in the current study because of the not so large size of the number of observations and the potential to present the short- and long-term interpretations. Accordingly, the ARDL estimation approach for equation (2) begins with

(4) $${lnOBESITY}_{t}=\beta_{0}+\sum_{k=1}^{p}\beta_{a}{lnOBESITY}_{t-k}+\sum_{k=0}^{q}\beta_{b,k}{lnINCOME}_{t-k}+\sum_{k=0}^{q}\beta_{c,k}{lnINCOME}_{t-k}^{2}+\sum_{k=0}^{r}\beta_{d,k}{lnGLOBAL}_{t-k}+\sum_{k=0}^{s}\beta_{e,k}\ln\;{GLOBAL}_{t-k}^{2}+\varepsilon_{t}$$

where $\beta_{0}$ presents the intercept and $\varepsilon_{t}$ is the error term at time t (where t = 1975, 1976, 1977, …, 2016). In addition, $\beta_{a}$, … $\beta_{e}$ are the respective elasticities for equation (4).

Consequently, the respective short-run and long-run deviations are presented with an associated adjustment parameter through the error correction model. To this end, the long-run coefficients is now empirically expressed in equation (5) below as:

(5) $${lnOBESITY}_{t}=\Psi_{0}+\rho_{1}{lOBESITY}_{t-1}+\rho_{2}\;\ln\;{INCOME}_{t-1}+\rho_{3}{lnINCOME}_{t-1}^{2}+\rho_{4}{lnGLOBAL}_{t-1}+\rho_{5}\;\ln\;{GLOBAL}_{t-1}^{2}+\sum_{k=1}^{p}\pi_{k}{lnOBESITY}_{t-k}+\sum_{i=0}^{q}\theta_{1,i}\Delta {lnINCOME}_{t-i}+\sum_{i=0}^{q}\theta_{2,i}\Delta {lnINCOME}_{t-i}^{2}+\sum_{j=0}^{s}\theta_{3,k}\Delta {lnGLOBAL}_{t-j}+\sum_{k=0}^{u}\theta_{4,k}\Delta \ln\;{GLOBAL}_{t-k}^{2}+\varepsilon_{t}$$

here Δ is a differenced operator while the long-run coefficients are obtained from $\sigma_{i}={\hat{\rho }}_{i}/\left(1-{\hat{\rho }}_{1}\right)$, i=1,2,3,4,5. Additionally, the error-correction term (ECT) is derived from $ect_{t-1}={lnOBESITY}_{t}-\ln\;{INCOME}_{t}-{lnINCOME}_{t}^{2}-\ln\;{GLOBAL}_{t}-\ln\;{GLOBAL}_{t}^{2}$. The coefficients $\sigma_{1},\sigma_{2},\sigma_{3},\sigma_{4},{and\;\sigma }_{5}$ are the long-run impacts of OBESITY, INCOME, square of INCOME, GLOBAL, and the square of GLOBAL on obesity. The use of ARDL is superior to previous basic method seen in extant literature like Analysis of variance (ANOVA) and chi-squares. The ARDL highlights both short-long run relationship simultaneously for ample policy crafting which is a strength of the method applied.

Similarly, the second model from equation (3) (robustness) that incorporates biological capacity (BCP) is estimated by employing the ARDL steps enumerated above. Therefore, the results of the aforesaid estimates are carefully presented in Table 2.

**Table 2:** ARDL cointegration estimation.

Bound Test				
F-statistic (k)	19.48495 (4)	Significance	I0 Bound	I1 Bound
		1%	3.74	5.06

	lnINCOME	lnINCOMEsq	GLOBAL	GLOBALsq	Adj. parameter
Long run	4.415	−0.218	0.316^a^	−0.002^b^	
Short run	0.472^a^	−0.021^c^	0.027^b^	−0.0001^b^	−0.095^a^
Diagnostic					
Breusch-Godfrey Serial Correlation Lagrange Multiplier Test (*p-value*): 0.285 (0.754)					
Heteroscedasticity Breusch-Pagan-Godfrey Test (p-value): 1.573 (0.174)					
Wald Test (Short run): F-statistic (*p-value*): 15.469 (0.000)					
Robustness estimate					
	lnINCOME	lnINCOMEsq	GLOBAL	LBCP	Adj. parameter
Long run	10.445^a^	−0.535^a^	0.049^a^	−1.858^b^	
Short run	0.817^a^	−0.038^a^	0.002	−0.022	−0.071^a^
Diagnostic					
Breusch-Godfrey Serial Correlation Lagrange Multiplier Test (p-value): 1.084 (0.353)					
Heteroscedasticity Breusch-Pagan-Godfrey Test (p-value): 1.279 (0.289)					

**Note**: The 1%, 5%, and 10% statistically significant levels are respectively a, b, and c.

## 4 Findings

As revealed in the result displayed in Table 2, the investigation revealed a positive and significant relationship between obesity and income, and a negative relationship between obesity and the square of income, especially in the short-run. The implication of the result is that the evidence of obesity Kuznets curve is valid vis-à-vis there is a significant and inverted U-shaped relationship between obesity and income in the United States. This is likened to the pioneering evidence of inequality-income nexus by Ref. [14]. Thus, the result posits that one of the pathways to achieve a less obese level and other weight-related issues in the United States is to ensure that per capita income is doubled over time. The evidence from this study aligns with the obesity Kuznets curve evidence in the most recent extant literature [22,34,47]. Specifically [22], found that adult obesity increases with a declining rate of the income level across the panel of 147 countries while [34] validated the obesity Kuznets curve for Oman, Saudi Arabia, Turkey, and the United Arab Emirates.

Moreover, this study further revealed the moderating role of globalisation in obesity and other weight-related issues for the United States. As opined in the study of [22], adult obesity prevalence is positively associated with political globalisation in the examined panel of 147 countries. Although [22] employed the political dimension of globalisation, our study considered the economic, social, and political dimensions of globalisation. Similarly, the result of [22], the current study affirms the same evidence of a significant and positive relationship between globalisation and obesity in both the short and long run at least at 95% credible interval. However, this study adds a different dimension to the relationship between obesity prevalence and globalisation by also unmasking the effect of the square of globalisation. As observed in Table 2, the square of globalisation is negatively correlated with obesity in both the short- and long-run at 95% credible interval. This implies that there is a statistically significant and inverted U-shaped relationship between globalisation and obesity in the United States.

Furthermore, the role of biological capacity (biocapacity) in obesity prevalence in the United States is observed to be negative and significant (see Table 2). Although biocapacity shows no statistically significant evidence of a negative relationship with obesity in the short run, the impact is negatively significant in the long run. In specific, a 1% increase in biocapacity is responsible to reduce obesity prevalence rate by 1.86% at a 5% statistically significant level. This result presents that the increase in the capacity of the ecological system to support or produce human basic needs will yield a decline in humans’ dependent on unnatural sources such as genetically modified food that are believed to have adverse health implications. Interestingly [48], revealed the ecological and metabolic food waste (MFW) effect of obesity in the seven Food and Agriculture Organisation (FAO) regions. Additionally [49], associated the adverse effect of the increasing ecological (carbon, water, and land use) footprint to the increase in obesity prevalence rate, thus yielding a decline in the biocapacity.

**Fig. 2:**
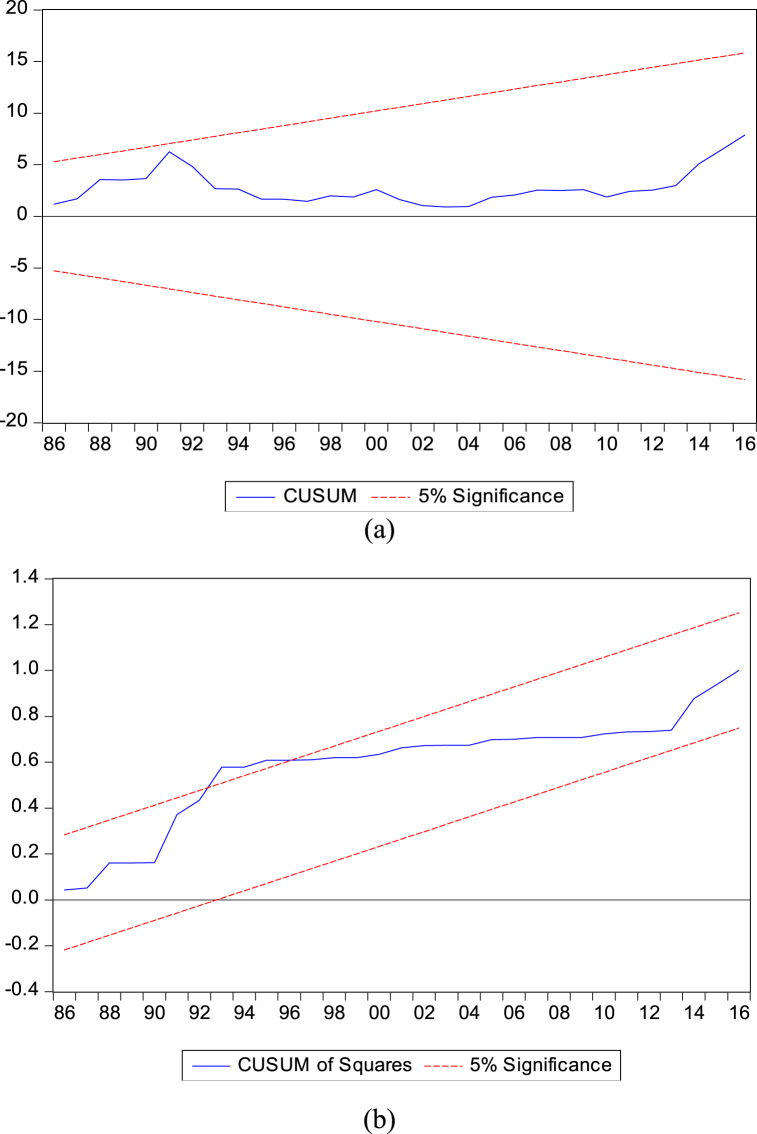
**(a**–**b)**: The model stability by CUSUM (a) and CUSUM of squares (b).

The diagnostic estimates from the two investigated models (equations (1), (3))) are desirable. In essence, there is no problem arising from heteroscedasticity and serial correlation in both cases since we fail to reject the respective null hypothesis. Interestingly, the models exhibit stability as observed from the cumulative sum and cumulative sum of squares for equation (1) in Fig. 2(a–b).

## 5 Conclusion and policy recommendation

With the Centres for Disease Control and Prevention (CDC) reporting that prevalence of obesity increased from 30.5% in the period 1999–2000 to 42.4% in 2017–2018 (Centres for Disease Control and Prevention, 2020), obesity and weight-related health issues are no less a concern in the United States. Importantly, in 2018, the CDC further noted that an obese American's medical expenditure is $1429 higher than those without weight-related health challenge. Motivated by this development, the current study examined the impact of income growth on obesity trend in the United States over the period of 1975–2016. In addition, the (moderating) roles of (social, economic, and political) globalisation and biological capacity are further evaluated in the study.

Therefore, the study revealed that increase in individual (per capita) income is a trigger for the trend of obesity in the United States, especially in the short run. However, a desirable relationship between obesity and income is significantly attainable, especially when income growth is doubled (square of income) thereby causing the obesity prevalence rate to begin to decline. This observation translates to the validity of the obesity Kuznets curve hypothesis vis-à-vis an inverted U-shaped relationship between income and obesity. In addition, social, economic and political globalisation also exhibit an inverted U-shaped relationship with obesity, thus suggesting that increased level of globalisation can mitigate the problem associated with weight-related health issues in the country. Moreover, biocapacity also plays a significant desirably mediating role both in the short and long run. In specific, there is a negative and significant relationship between biocapacity and obesity prevalence with an elasticity of 0.02 and 1.85 in the short- and long-run, respectively. Although this study is limited in that it is a national case and does not provide state-specific relevant information, however, there are deducible national policy insights from the result of the investigation.

### 5.1 Policy

Considering the enormous challenge associated with weight-related health issues in the United States, this study offers useful policy for government and health agencies in the country. The central and governments at state levels should continue to implement welfare and socioeconomic programmes that potentially improve the income status of the citizens. Additionally, more proactive measures that potentially improve the Earth's biocapacity, such as preventive measures against wildfire and other environmental hazard, should be further prioritized and considered when drafting policy-related instrument across the sectors of the economy. The government can also reconsider some of its foreign policy, especially from the standpoint of (socioeconomic and political) globalisation such that trade, health and related policies are commensurate with the drive to reduce adult obesity prevalence in the country. Lastly, policies that guides production and especially the value chain of all agricultural-related aspects could be revisited in order to further address the urgency to significantly reduce obesity prevalence rate in the United States.

## Author contribution statement

Gloria Nnadwa Alhassan: Conceived and designed the experiments, conceptualization, Writing - review & editing. Andrew Adewale ALOLA: Conceived and designed the experiments, conceptualization, Contributed to data and empirical tool, Data curation; Analyzed and interpreted the data, Investigation; Writing - review & editing. Festus Victor BEKUN: Conceived and designed the experiments, conceptualization, Contributed to data and empirical tool, Analyzed and interpreted the data, Investigation; Writing - review & editing.

## Data availability statement

Data will be made available on request.

## Additional information

No additional information is available for this paper.

## Funding

No funding has been received toward the research.

## Ethics approval and consent to participate

Not applicable.

## Consent for publication

Not applicable.

## Declaration of competing interest

The authors declare that they have no known competing financial interests or personal relationships that could have appeared to influence the work reported in this paper.

## Figures and Tables

**Fig. 1:**
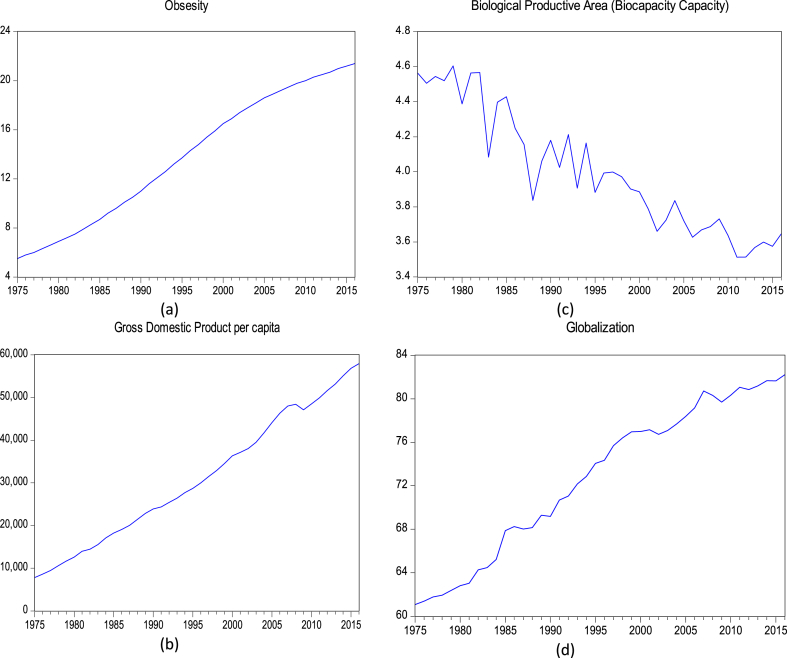
(a–d): The trend of the examined variables.

## Appendix

### List of abbreviations

WHO: World Health Organisation
BMI: mass index
NCD: non-communicable disease
GDP: Gross Domestic Product
SDG: Sustainable Development Goals
CDC: Centres for Disease Control and Prevention
ARDL: Autoregressive distributed lag method
